# Association between single nucleotide polymorphism of IL15RA gene with susceptibility to ossification of the posterior longitudinal ligament of the spine

**DOI:** 10.1186/s13018-014-0103-6

**Published:** 2014-11-12

**Authors:** Qiang Guo, Shou-Zheng Lv, Shu-Wen Wu, Xu Tian, Zhi-Yong Li

**Affiliations:** Department of Spinal Surgery, Tianjin Baodi Hospital, No 8 Guangchuan Street, Baodi District Tianjin, 301800 China; Department of Orthopaedics Institute, Tianjin Hospital, 406 Jiefang Nan Street, Hexi District Tianjin, 300211 China

**Keywords:** Ossification of the posterior longitudinal ligament, Single nucleotide polymorphism, Interleukin 15 receptor alpha, Association study

## Abstract

**Background:**

Previous studies reported the association between single nucleotide polymorphism (SNP) of IL15 receptor alpha (IL15RA) gene with susceptibility to ossification of the posterior longitudinal ligament of the spine (OPLL). However, the results were still in controversy. Therefore, the purpose of the present study was to investigate the association between SNPs of IL15RA gene with susceptibility to OPLL in a Chinese Han population.

**Methods:**

A total of 235 OPLL patients and 250 age-matched healthy controls were recruited. All the subjects were genotyped using the PCR (polymerase chain reaction)-based invader assay. A case–control study was performed to define the contribution of rs2228059 and rs2296139 to predisposition of OPLL. We also performed subgroup analysis according to the different gender.

**Results:**

A significant association of rs2228059 with OPLL was observed in the Chinese Han population (*p* <0.001, OR = 1.63, 95% CI = 1.26–2.11). The subgroup analysis showed that there was a significant association between the allele frequency of rs2228059 and the susceptibility of OPLL in males (*p* = 0.002, OR = 1.72, 95% CI = 1.23–2.42). However, there was no significant association between SNP of rs2296139 and susceptibility to OPLL.

**Conclusions:**

The present study demonstrates that the SNP of rs2228059 in IL15RA gene is associated with susceptibility to OPLL in a Chinese Han population, especially in males.

## Introduction

Ossification of the posterior longitudinal ligament of the spine (OPLL) is an abnormal finding most prevalent in the Asian population, characterized by a pathological ectopic bone formation in the posterior longitudinal ligament [1]. The primary clinical feature of OPLL runs within the spinal canal; patients subsequently develop compressive myelopathy or radiculopathy. OPLL has been shown to lead to myelopathy in approximately 17% of those affected [2]. Because OPLL is slowly progressive, clinical manifestation ranges from radiological ossification without symptoms to severe myelopathy that leads to unrecoverable tetraplegia. Clinical severity correlates with the size and extent of the ossified lesion. Most patients who have significant symptoms require surgical treatment, although the postoperative courses are often unsatisfactory.

OPLL is thought to be a multifactorial disease [3]. Patients tend to develop systemic hyperostosis and have high bone mass, which may lead to ectopic ossification beyond the spine, suggesting the presence of systemic factors that confer susceptibility to the disease [3]. The involvement of genetic factors has been confirmed as well. Moreover, genetic factors played an important role in the development of such disease. These complex genetic contributors included collagen alpha 2 (VI), collagen alpha 2 (XI), nucleotide pyrophosphatase, leptin receptor, estrogen receptor, and bone metabolism regulatory cytokines such as bone morphogenetic protein 2, transforming growth factor beta 1, and interleukin (IL)-1 beta [4-9].

IL15 receptor alpha (IL15RA) mediates IL15 function [10]. IL15 is reported to have an activity to stimulate the differentiation of osteoclast progenitors into osteoclast [11]. IL15RA is polymorphic and has been reported that IL15RA polymorphisms are associated with muscle, bone, responses of resistance exercise training, and body composition [12-14]. Kim et al. [15] firstly reported that IL15RA polymorphism may be associated with the susceptibility of OPLL in Korean population. However, there was no gender-specific haplotype association of single nucleotide polymorphisms (SNPs) in the IL15RA with OPLL reported. Therefore, the purpose of the present study is to investigate the association between SNPs of IL15RA gene with susceptibility to ossification of the posterior longitudinal ligament of the spine in a Chinese Han population.

## Materials and methods

### Subjects

A total of 485 subjects in the Chinese Han population were studied. Two hundred thirty-five OPLL patients (161 males and 74 females) were recruited from the Spine Surgery, Baodi Renmin Hospital of Tianjin Medical University between May 2009 and August 2013. The diagnosis of OPLL was made based on radiographic examinations conducted by experienced spinal surgeons. Patients with metabolic or endocrinologic disorders known to be associated with secondary OPLL including acromegaly, hypophosphatemic rickets, or osteomalacia and hyperparathyroidism were excluded. The range of ossified lesion was evaluated by the total number of ossified vertebrae apparent on lateral radiographs of the cervical, thoracic, and lumber spine. OPLL severity was classified by clinical or MR evidence of myelopathy. The age-matched control group did not have OPLL or other systemic diseases. This study was approved by the ethics review committee of the Medical Research Institute, Tianjin Medical University, and written informed consent was obtained from all subjects.

### SNP selection

Two SNPs with greater than 0.1 heterozygosity among SNPs located in the exon regions of the IL15RA gene (rs2296139, exon 2, Thr73Thr; rs2228059, exon 4, Asn182Thr) (http://www.ncbi.nlm.nih.gov/SNP, SNP database, Build 130) were selected.

### Genotyping

Genomic DNA was extracted from peripheral blood leukocytes using genomic DNA isolation kits (Promega, Madison, WI) according to the manufacturer’s instructions. The primers, probes, and reaction conditions were available upon request. SNPs were genotyped by the PCR (polymerase chain reaction)-based invader assay (Third Wave Technologies) using ABI 7900 (Applied Biosystems, Foster City, CA, WI) [16]. Genotyping was done by a laboratory personnel blinded to subject status. Of the samples, 10% were tested twice to validate the genotyping results with 100% reproducibility. Two authors independently reviewed the genotyping results, data entry, and statistical analysis.

### Statistical analysis

Standard *X*^2^ analysis of contingency tables was used to examine differences of allelic frequencies and genotype distributions between OPLL patients and controls. Hardy–Weinberg equilibrium (HWE) for two SNPs was assessed using a SNPStats (http://bioinfo.iconcologia.net/index.php) [17]. Odds ratio (OR) and 95% confidence interval (CI) were calculated using the reported risk allele. Statistical significance was considered at *p* <0.05.

## Results

A total of 485 subjects (235 cases and 250 controls) were successfully genotyped and subjected to statistical analysis (Table 1). The distributions of the alleles and genotypes for rs2228059 and rs2296139 were presented in Tables 2 and 3. In the control group, the genotype frequencies for two SNPs were in Hardy–Weinberg equilibrium (rs2228059, *p* = 0.51; rs2296139, *p* = 0.55).

**Table 1:** **Characteristics of patients**

**Parameters**	**Values**
Number of patients	235
Sex	
Female	74
Male	161
Mean age (range)	65 (49–82)
Number of vertebral with OPLL	
<3	29
4–7	145
>7	61

*OPLL,* ossification of the posterior longitudinal ligament.

**Table 2:** **Allele and genotype frequencies of rs2228059 in OPLL in a Chinese Han population**

	**Genotype (%)**			***p*** **value**	**Allele (%)**		***p*** **value**	**OR (95% CI)**
	**AA**	**AC**	**CC**		**A**	**C**		
Total cases	93 (39.6%)	116 (49.4%)	26 (11.1%)		302 (64.3%)	168 (35.7%)		
Total controls	65 (26.0%)	132 (52.8%)	53 (21.2%)	<0.001	262 (52.4%)	238 (47.6%)	<0.001	1.63 (1.26, 2.11)
Male cases	70 (43.5%)	72 (44.7%)	19 (11.8%)		212 (65.8%)	110 (34.2%)		
Male controls	34 (27.2%)	64 (51.2%)	27 (21.6%)	<0.001	132 (52.8%)	118 (47.2%)	0.002	1.72 (1.23, 2.42)
Female cases	23 (31.1%)	44 (59.5%)	7 (9.4%)		90 (60.8%)	58 (39.2%)		
Female controls	31 (24.8%)	68 (54.4%)	26 (20.8%)	0.060	130 (52.0%)	120 (48.0%)	0.088	1.43 (0.95, 2.17)

*OPLL,* ossification of the posterior longitudinal ligament, *OR,* odds ratio.

**Table 3:** **Allele and genotype frequencies of rs2296139 in OPLL in a Chinese Han population**

	**Genotype (%)**			***p*** **value**	**Allele (%)**		***p*** **value**	**OR (95% CI)**
	**GG**	**GA**	**AA**		**G**	**A**		
Total cases	97 (41.3%)	103 (43.8%)	35 (14.9%)		297 (63.2%)	173 (36.8%)		
Total controls	100 (40.0%)	113 (45.2%)	37 (14.8%)	0.491	313 (62.6%)	187 (37.4%)	0.849	1.03 (0.79, 1.33)
Male cases	61 (37.9%)	77 (47.8%)	23 (14.3%)		199 (61.8%)	123 (38.2%)		
Male controls	53 (42.4%)	59 (47.2%)	13 (10.4%)	0.198	165 (66.0%)	85 (34.0%)	0.301	0.83 (0.59, 1.18)
Female cases	28 (37.8%)	36 (48.6%)	10 (13.5%)		92 (62.2%)	56 (37.8%)		
Female controls	51 (40.8%)	60 (48.0%)	13 (10.4%)	0.404	162 (64.8%)	86 (35.2%)	0.526	0.87 (0.57, 1.33)

*OPLL,* ossification of the posterior longitudinal ligament, *OR,* odds ratio.

The frequencies of AA, AC, and CC genotypes (rs2228059) were 39.6%, 49.4%, and 11.1% in the OPLL group and were 26.0%, 52.8%, and 21.2% in the control group, respectively. The genotype analysis of rs2228059 showed significant association between OPLL and control groups. The allele frequency of rs2228059 was also significantly associated with the risk of OPLL (*p* <0.001, OR = 1.63, 95% CI = 1.26–2.11). This suggests that the allele (A) of rs2228059 may be associated with an increased risk of OPLL. The significant association between an allele frequency of rs2228059 and susceptibility of OPLL was also found in males (*p* = 0.002, OR = 1.72, 95% CI = 1.23–2.42) (Table 2).

The frequencies of GG, GA, and AA genotypes (rs2296139) were 41.3%, 43.8%, and 14.9% in the OPLL group and were 40.0%, 45.2%, and 14.8% in the control group, respectively. The genotype analysis of rs2296139 showed no significant association between OPLL and control groups. The allele frequency of rs2296139 was not significantly associated with the risk of OPLL (*p* = 0.849, OR = 1.03, 95% CI = 0.79–1.33) (Table 3).

## Discussion

OPLL is a complex trait, and complicated etiologies need to be considered for the understanding of the underlying pathogenesis, in which both genetic and environmental factors play mutual roles. Genome-wide association study (GWAS) is the most robust approach to identify predisposition genes for common diseases and complex traits [18], and it has been increasingly used to study genetic predisposition in OPLL that is regarded as one of the most common complex genetic disorders of the musculoskeletal system. However, this method may produce spurious association [19,20]. Therefore, replications of the associations in different ethnic groups and studies with large sample sizes are important to confirm the results of GWAS [21].

IL15RA gene is located on chromosome 10p15-p14 and polymorphic. IL15RA has pleiotropic roles on immune development and function [22]. This cytokine receptor binds IL15 with high affinity. IL15RA is thought to play a crucial role in IL15 signaling [11]. Several studies reported that IL15 and IL15RA have potential role in bone remodeling. Petrovic-Rackov et al. [23] reported that IL15 level in serum and synovial fluid of patients with rheumatoid arthritis was significantly higher than those with osteoarthritis and correlates with a disease activity. Ogata et al. [10] reported that IL15 can stimulate osteoclastogenesis through IL15RA.

Furthermore, several studies reported that there were genetic associations of IL15RA gene polymorphisms with bone volume, muscle strength and volume, and metabolic syndrome [12-14]. Although only one study has suggested that IL15RA are associated with the susceptibility of OPLL in Korean population [15], genetic association is not yet known in other population or reaffirmed. Therefore, the present study investigated the genetic association between IL15RA polymorphisms and the susceptibility to OPLL in Chinese Han population and evaluated the difference between male and female.

The present study shows that the coding SNP (rs2228059) in the IL15RA gene contributes to the susceptibility of OPLL in a Chinese Han population. However, the SNP of rs2296139 is not associated with the susceptibility of OPLL. The present results are in consistency with the previous study which conducted in a Korean population [15]. Because OPLL has male predominance of 2:1 to 4:1, we also divided the OPLL group into two subgroups (male or female) and examined the relationship between gender difference and IL15RA polymorphisms. The results suggest that SNP of rs2228059 was associated with OPLL in males.

Pistilli et al. [12] found that the presence of the A-allele of the rs2228059 IL15RA SNP was associated with a greater baseline whole muscle volume in males. The study also demonstrated that the A-allele of rs2228059 was associated with a greater baseline cortical bone volume in males. The C-allele of rs2228059 may be associated with a lower baseline whole muscle volume and lower baseline cortical bone volume. The above results suggested that the SNP of rs2228059 may be contributed to the pathogenesis of OPLL. Pistilli et al. [12] also reported the difference between male and female. In the present study, gender difference was also observed. In male patients, genotype of rs2228059 was associated with OPLL, and the allele frequency of rs2228059 was also significantly associated with OPLL. However, there is no significant association in female OPLL. The results indicate that genetic factors involved in IL15RA play an important role in the etiology of OPLL in males.

Although there was no significant association found in female, several factors may contribute to this discrepancy. Firstly, sex hormones or other gender-specific factors may be more important than the genetic variation in IL15RA in females. It is well-established that estrogen plays a crucial role in the maintenance of a bone mineral, because bone loss is frequently observed in postmenopausal women. Estrogen has a profound effect on bone development and remodeling [24,25]. It should be emphasized that OPLL patients generally show a tendency of having high bone mineral density; therefore, they are in a hyperostotic state, regardless of age and sex. Secondly, most of the genetically high-risk women may escape manifestations of OPLL because of the tendency toward bone loss caused by estrogen decrease after menopause. Lastly, the limited sample size in female may lower the statistical power and will decrease the confidence in explanation for this discrepancy. The above statements may explain, in part, the gender-specific genetic associations of IL15RA and differences in OPLL prevalence between males and females.

IL15RA protein (Q13261) is constituted in 267 amino acids (AAs) and consists of several parts including the signal peptide (1–30 AAs), Sushi domain (31–95), extracellular topological domain (31–205), helical transmembrane region (206–228), and cytoplasmic topological domain (229–267) (http://www.uni-prot.org/uniprot/). A missense SNP of rs2228059 of the IL15RA gene is located at the extracellular topological domain. Anderson et al. [26] demonstrated that the allelic variation of rs2228059 results in the difference of IL15RA binding affinity. Therefore, the altered IL15 binding affinity by the A-allele of rs2228059 may be contributed to the development of OPLL.

Despite the current evidence suggesting SNP of rs2228059, IL15RA play an important role in the etiology of OPLL in a Chinese Han population especially in males; the following significant limitations persist in the present study: (1) We were unable to conduct subgroup analysis for every confounding factor, including severity of OPLL and age; (2) Efficacy of statistics may be further improved by including more samples in the future; (3) The effect of genetic and environmental interactions cannot be addressed completely in the present study; (4) Considering the difference in ethnicity, the present results need to be replicated in other populations.

## Conclusions

The present study demonstrates that the SNP of rs2228059 in IL15RA gene is associated with susceptibility to OPLL in a Chinese Han population, especially in males. However, there was no significant association between the SNP of rs2296139 in IL15RA gene and susceptibility to OPLL. Due to the finite evidence currently available in Chinese Han population, further studies with large sample size or other ethnicity are still required.
